# Contrast and Conflict in Dutch Vowels

**DOI:** 10.3389/fnhum.2021.629648

**Published:** 2021-06-07

**Authors:** Nadine P. W. D. de Rue, Tineke M. Snijders, Paula Fikkert

**Affiliations:** ^1^Centre for Language Studies, Radboud University, Nijmegen, Netherlands; ^2^Max Planck Institute for Psycholinguistics, Nijmegen, Netherlands; ^3^Donders Institute for Brain, Cognition and Behaviour, Radboud University, Nijmegen, Netherlands

**Keywords:** perceptual asymmetry, vowels, Dutch, MMN, conflict, phonological contrasts

## Abstract

The nature of phonological representations has been extensively studied in phonology and psycholinguistics. While full specification is still the norm in psycholinguistic research, underspecified representations may better account for perceptual asymmetries. In this paper, we report on a mismatch negativity (MMN) study with Dutch listeners who took part in a passive oddball paradigm to investigate when the brain notices the difference between expected and observed vowels. In particular, we tested neural discrimination (indicating perceptual discrimination) of the tense mid vowel pairs /o/-/ø/ (place contrast), /e/-/ø/ (labiality or rounding contrast), and /e/-/o/ (place and labiality contrast). Our results show (a) a perceptual asymmetry for place in the /o/-/ø/ contrast, supporting underspecification of [CORONAL] and replicating earlier results for German, and (b) a perceptual asymmetry for labiality for the /e/-/ø/ contrast, which was not reported in the German study. A labial deviant [ø] (standard /e/) yielded a larger MMN than a deviant [e] (standard /ø/). No asymmetry was found for the two-feature contrast. This study partly replicates a similar MMN study on German vowels, and partly presents new findings indicating cross-linguistic differences. Although the vowel inventory of Dutch and German is to a large extent comparable, their (morpho)phonological systems are different, which is reflected in processing.

## Introduction

There is considerable acoustic variation in natural speech, making recognition of spoken words or even single vowels rather complex. For word recognition, it is important to perceive meaningful differences (i.e., phonological features), which are contrastive and to some extent predictable. The phonological underlying representations of words, and indeed phonemes, in our mental lexicon are made up of phonological features that play a crucial role in recognition. A vital issue in phonology is what information these representations contain and how they enable us to recognize and produce words the way we do. Some theories assume rich phonetic detail to be part of stored representations (e.g., Johnson, 1997; Goldinger, 1998; Bybee, 2001; Pierrehumbert, 2002; Polka and Bohn, 2003, 2011; Masapollo et al., 2017a,b), while others only assume the essential features needed to differentiate between lexical contrasts (e.g., Chomsky and Halle, 1968; Archangeli, 1988; Lahiri and Reetz, 2002; Dresher, 2009). One model that makes very explicit assumptions as to which phonological features are stored in the underlying representations in the mental lexicon is the Featurally Underspecified Lexicon (FUL) model (e.g., Lahiri and Reetz, 2002, 2010; Lahiri, 2018).

FUL aims to define and regulate a set of features which can cover the typology of all possible contrasts and alternations in the languages of the world (Lahiri, 2018). In addition, it is able to account for acquisition and language processing. The model assumes privative phonological features (i.e., presence or absence of features), and furthermore assumes only contrastive features to be stored in mental representations. For the contrasts under investigation in this paper, the features [LABIAL], [CORONAL], and [DORSAL] – which reside under the ARTICULATOR node – are in focus. [CORONAL] is assumed to be the universal default, and hence is underspecified in the underlying representation. In FUL, features are identical for vowels and consonants. There are no feature dependencies, and features underneath each node are mutually exclusive, although in the current model of FUL [LABIAL] can combine with [CORONAL] and [DORSAL] for vowels, an assumption we will further address in the discussion. Furthermore, the model assumes that listeners extract features from the acoustic signal which are mapped onto the features of the underlying representations in the mental lexicon. These perceived surface features can *match*, *mismatch* or *no*-*mismatch* with the underlying features. Matches and mismatches require features to be detected from the input and present in the underlying representation. A no-mismatch occurs when an extracted surface feature is underspecified in the underlying representation (e.g., default). This ternary mapping procedure predicts specific asymmetries in a Mismatch Negativity (MNN) experiment. A mismatch is predicted to result in a larger neural discrimination difference between two sounds than a no-mismatch, reflected by an enhanced or earlier MMN (Näätänen, 2001), indicating perceptual discrimination.

**FIGURE 1:**
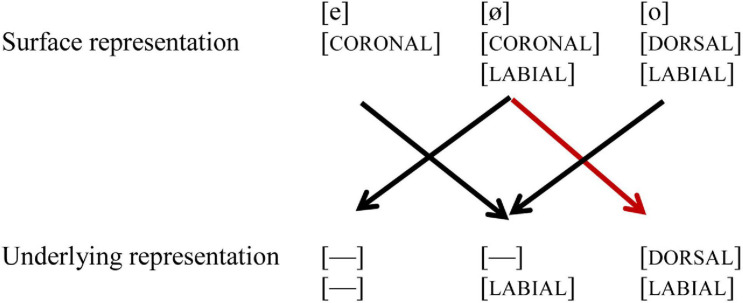
Black lines indicate a no-mismatch, the red line indicates a mismatch. [—] indicates underspecification.

In their seminal MMN study, Eulitz and Lahiri (2004) showed that German listeners perceived vowel contrast asymmetrically, and argued that this asymmetry is due to phonological underspecification of coronal place of articulation. While the acoustic difference between the mid vowels /e, ø/ on the one hand, and /o, ø/ on the other is similar, their phonological representations differ, as shown in (1): while /e, ø/ are underspecified for place of articulation in the underlying representation, indicated by [—], /o/ is specified as [DORSAL]. Furthermore, /o, ø/ both are specified for [LABIAL]. However, the underspecified feature [CORONAL] can still be extracted from the acoustic signal, and thus be part of the surface representation, just like [LABIAL] and [DORSAL]. When in an oddball paradigm the standard (indicated by //) is presented, the underlying representation of the standard is pre-activated. Upon hearing a deviant (indicated by []), the surface representation of the deviant is mapped onto the pre-activated underlying representation of the standard. When the standard is /o/, the coronal place of articulation of the deviant [ø] will lead to a mismatch (marked with a red line), but the reverse will not, as the perceived [DORSAL] feature of [o] will form a no-mismatch with the underspecified [CORONAL] in the underlying representation of /ø/, as shown in Figure 1.

Based on these perceptual asymmetries, Eulitz and Lahiri (2004) argue that coronal is underspecified in German. These perceptual asymmetries of place of articulation (in particular [CORONAL] and [DORSAL]) have also been found in various subsequent studies for vowels in isolation, as well as in words and non-words (Cornell et al., 2011). The role of [LABIAL] is not much discussed in the paper, which is something we will pay attention to in the current study. The contrast /e, ø/ shares the same place of articulation [CORONAL], but differs in the value [LABIAL]: /ø/ is specified for [LABIAL], while /e/ is not. The contrast /o, ø/ differs in place of articulation, /ø/ being [CORONAL] on the surface, while /o/ is [DORSAL], but they share the feature [LABIAL] (see Figure 1).

Like German, Dutch has a three-way contrast between /e, ø, o/, and this suggests that the same underlying representations may be at stake. If so, we should replicate the perceptual asymmetries in German for Dutch, which is our first aim. Yet, looking at the linguistic system as a whole, the nature of the front rounded vowels may be different in Dutch and German. In German, many front rounded vowels arise because of morphological umlaut, which fronts back vowels in certain plurals, diminutives, adjectival and verbal forms. For example, German has the singular-plural alternation *Vogel – Vögel* “bird(s),” whereas Dutch has *vogel – vogels*, without vowel alternation. Although the original motivation for Umlaut was phonological (Twadell, 1938), in modern German, Umlaut is no longer phonologically transparent, and has become a morphological rule (Wiese, 1996). Consequently, the /ø/ predominantly occurs in morphologically derived contexts, and might not be part of the underlying vowel inventory of German. Scharinger (2009) has argued that the underlying representations of stems that may alternate between front rounded and back rounded vowels in morphologically derived context may differ from stems that do not alternate. Non-alternating stems with front rounded vowels are thus much less frequent in German than in Dutch. Modern Dutch does not have morphological Umlaut, and front rounded vowels are truly part of the Dutch vowel inventory. The second aim of this paper is to investigate whether this difference between German and Dutch is reflected in differences in phonological processing.

Another difference between the German and Dutch vowels /e, ø, o/ is that the Dutch realization of vowels can be considered semi-diphthongized (Adank et al., 2004), while the German ones have more stable formant frequencies. Phonologically, however, these tense mid vowels are not diphthongs in Dutch; the formant transition is not obligatory. Diphthongization strengthens place features toward the end of vowels, as front vowels become even more front, impacting the second formant (F2), which is the acoustic cue for the front-back dimension. Furthermore, vowels may become higher toward the end, acoustically reflected by a lower first formant (F1). Although the diphthongization may not immediately affect the phonological representations of Dutch vowels, it may make place of articulation contrasts larger, and hence more difficult to find asymmetries. No effect is expected on labiality. Plots and Tables of the Dutch and German vowel formats are given in Supplementary Appendix 3.

The current paper therefore replicates the study of Eulitz and Lahiri (2004) and investigates the processing of the same three-way vowel contrast in Dutch. Similar to Eulitz and Lahiri (2004), we measured the MMN in an electroencephalography (EEG)-experiment, but in Dutch listeners. Like in the original study, we tested discrimination of tense mid vowels /e/, /ø/, and /o/ in isolation in a passive oddball paradigm. We predicted to find the same perceptual asymmetry for place (i.e., in the vowel pair /o/-/ø/ we expect to find a larger MMN when [ø] is the deviant, than when [o] is the deviant), supporting underspecification of [CORONAL]. We are less certain about the predictions in the vowel pair /e/-/ø/, which are both coronal, but differ in labiality (rounding), as these are not addressed in Eulitz and Lahiri (2004). There are two reasons to want to investigate potential asymmetries for these vowels. The first reason is that for vowels [LABIAL] is not mutually exclusive with [CORONAL] or [DORSAL], while the latter two are mutually exclusive. This suggests that [LABIAL] has a different status. The second reason is that, although Eulitz and Lahiri do not report an asymmetry for the pair /e/-/ø/, it is not entirely straightforward how the model could account for the discrimination of these vowels: the perceived [CORONAL] of /e/ does not seem to mismatch with [LABIAL] of /ø/ in this case, as /ø/ itself is coronal. This too suggests a different status of [LABIAL]. Hence, investigating listeners’ brain responses to this contrast may provide new insights into the nature of the representations of these vowels.

## Materials and Methods

We replicated the German electroencephalographic (EEG) study by Eulitz and Lahiri (2004) in Dutch. We measured differences in brain responses to three tense mid vowels /e, ø, o/ in Dutch by means of a MMN experiment, using a passive oddball paradigm. The three vowels form three vowel pairs, for which we tested both directions of discrimination. This way, we aim to gain insight in the underlying representations of these sounds with respect to place of articulation and labiality (also known as rounding).

This experiment was conducted at the Donders Centre for Cognitive Neuroimaging in Nijmegen (Netherlands). The study was approved by the local ethics committee “Commissie Mensgebonden Onderzoek” (CMO) Arnhem-Nijmegen, Netherlands, under the general ethics approval (Imaging Human Cognition, CMO 2014/288), and the experiment was conducted in accordance with these guidelines.

### Participants

Seventeen right-handed adult native Dutch speakers (10 female, aged 18–30) were included in the final analysis. Participants were recruited using the Radboud Research Participation System (SONA Systems Ltd.). Subjects grew up as monolingual Dutch speakers at least until the age of twelve. Dialect speakers were excluded from participation, as several Dutch dialects have morphological Umlaut, comparable to German. Participants had normal hearing, did not suffer any language or speech impediments (e.g., dyslexia, cleft palate, DLD, etc.), and had not received speech therapy at present or in the past. They had no background in linguistics. Prior to participating, subjects signed an informed consent and were screened for EEG-compatibility (e.g., with respect to claustrophobia or epilepsy). Subjects received a financial reimbursement or study course credits.

Power analysis was done in G^∗^power, based on the values for the F-test for the mean amplitude of Fz reported in Eulitz and Lahiri (2004), using a partial omega squared of 0.245 [based on *F*(1,11 = 5.21), see Lakens (2013)]. With the resulting effect-size *f*(U) of 0.57, for a power of 0.8 fourteen participants were required as a minimum for the current study. We used six versions of the experiment to counterbalance order of presentation (each testing all conditions), and we preferred to test each version with an equal number of participants. We therefore aimed for eighteen participants – three participants per version.

In total, twenty-four participants completed this EEG study. Six subjects were excluded due to technical errors (4 subjects), the use of antidepressants (1 subject), and too noisy EEG data (1 subject). These subjects were replaced to get to the aimed 18 subjects. One final subject was excluded due to the failure to show an MMN response to any condition nor overall, resulting in 17 included participants in the final analysis.

### Task and Apparatus

Subjects participated in a passive oddball paradigm. Their electrical brain activity was recorded by means of EEG while they listened to streams of vowels in isolation. Stimuli were presented using Presentation Software (version 18.2 02.18.16). The complete EEG-recording took roughly 1.5 h. In every block, one vowel category occurred frequently (the *standard* stimuli), interspersed with tokens of a vowel category that occurred infrequently (the *deviant* stimuli). For instance, participants heard /e/ as a standard and [o] as a deviant sound in one block. Apart from staying awake, there was no explicit task or overt response. During the experiment, participants were seated in front of a computer screen (Benq XL2420Z – 24 inch) in a sound-attenuated booth. Participants were instructed to sit as still and relaxed as possible, to reduce movement artifacts, eye movements and blinks as much as possible. Auditory stimuli were presented through circumaural passive noise canceling Sennheiser headphones. Participants watched a silent movie which kept them engaged and awake for the duration of the entire experiment. The film was presented at screen center with half screen width to minimize saccades. Viewing distance was approximately 100 cm.

### Design and Procedure

We tested discrimination of three tense mid vowels /e, ø, o/, which were presented as vowels in isolation. These three vowel categories can be combined into three different vowel pairs (see Table 1). For each vowel pair, two conditions were tested, referring to different directions of change: e.g., for vowel pair /e, ø/, in one direction of change /e/ served as a standard and [ø] as a deviant, while in the opposite direction of change /ø/ is the standard and [e] serves as the deviant. With two directions of change for all three vowel pairs, this results in six conditions. Each condition was tested in a separate block. Each participant was tested on all six conditions.

**TABLE 1:** Overview of experimental conditions.

Vowel pair	Condition	Standard	Deviant	Contrastive feature(s)
/ø/-/o/	[ø]/o/	/o/	[ø]	Place
	[o]/ø/	/ø/	[o]	
/e/-/ø/	[e]/ø/	/ø/	[e]	Labiality
	[ø]/e/	/e/	[ø]	
/e/-/o/	[e]/o/	/o/	[e]	Place and labiality
	[o]/e/	/e/	[o]	

*For each *vowel pair* two conditions are tested in order to test two directions of discrimination. During testing, surface features of the deviant are mapped onto the underlying features of the standard. A vowel in // represents the standard sound. Vowels in square brackets [] represent the deviant vowel. E.g., condition [e]/o/ measures the response to vowel [e] as a deviant in a stream of /o/-stimuli.*

In every block, 1,000 vowels were presented in a passive oddball paradigm, with 85% standards and 15% deviants. An inter stimulus interval of 700 ms was used. Note that due to misinterpretation this ISI is longer than the 500 ms ISI in Eulitz and Lahiri (2004). We used six different orders of blocks – counterbalanced across subjects. Two successive blocks never had the same standard vowel. Each block lasted for ∼15 min. Participants were free to take a break in between the blocks. Participants themselves indicated when they were ready to start the next block. For each of the six different block orders, different pseudo-randomized within-block stimulus lists were used. Pseudo-randomized stimulus lists ensured that every block started with at least three tokens of the standard, and a deviant was always followed by at least two and maximum eleven standards before another deviant was presented. Deviants occurred unpredictably.

### Stimuli

Our standard and deviant stimuli were three different tokens of the three Dutch vowels [e] as in *zeef* (“sieve”), [ø] as in *deuk* (“dent”), and [o] as in *poot* (“paw”/“leg”), spoken by a male Dutch native speaker. By using three tokens of each vowel, acoustic variability was introduced to simulate more natural speech perception conditions and to force the processing system to map the incoming acoustic signals onto more abstract representations, rather than focus on properties unimportant in verbal processing (Eulitz and Lahiri, 2004). The vowels were recorded in isolation, and in /hV/ context, where the /h/ was spliced off. For more details regarding stimulus creation, see Supplementary Appendix 1.

Stimuli had a duration of 200 ms with 50 ms offset ramps. F0 was similar (∼112 Hz) in all vowel categories. dBA values measured at the headphones for all vowels were within a 1 dB range of 64 dB. Since different vowel categories have different frequency characteristics, which could lead to differences in perceptual loudness, we assessed differences in perceptual loudness in a behavioral pretest. In addition, we assessed the degree of within-category variation by means of a pretest as well. Both these pretests are reported in Supplementary Appendix 2.

**FIGURE 2:**
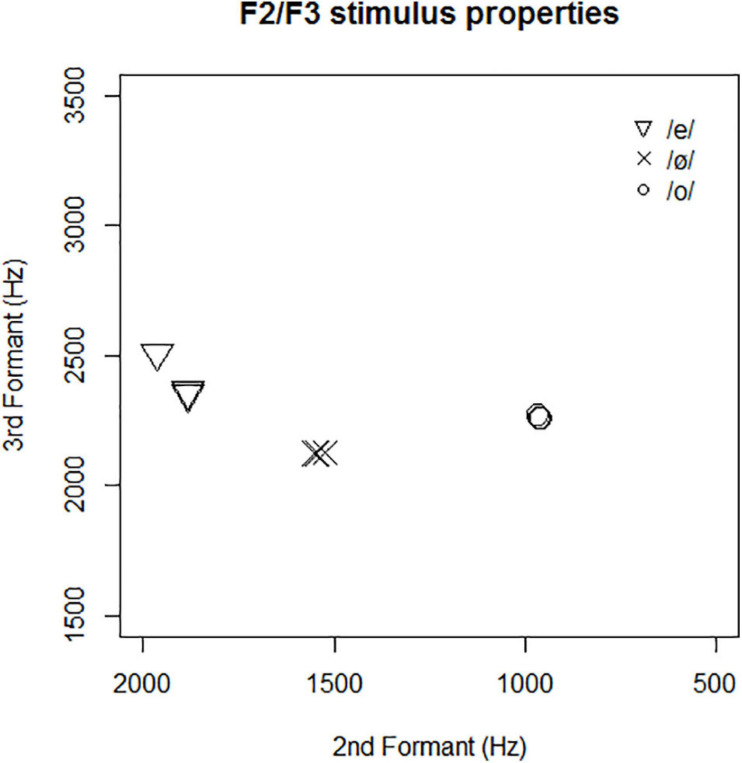
Each of the three tokens per vowel category plotted in F2/F3 space.

The vowel categories mainly differ with respect to F2 and F3, which are related to place and labiality features. In Figure 2, the three tokens of each of the three vowel categories are placed in the F2/F3 vowel space. Figure 2 shows that the acoustic distance between [e] and [ø] on the one hand, and between [o] and [ø] on the other, was quite similar. Acoustic stimulus characteristics are reported in more detail in Supplementary Appendix 3.

### Electroencephalography Recording

Electroencephalography data was recorded in Brain Vision Recorder (Brain Products GmbH, Munich, Germany) with 64 active electrodes (ActiCAP, equidistant – Brain Products) against left mastoid as an online reference, using a sampling rate of 500 Hz. Eye movements and blinks were tracked by four EOG (electrooculography) electrodes: one above and one below the left eye tracking vertical movement and blinks, and one left of left eye and one right of right eye tracking horizontal eye movements. Impedance levels of <20 kΩ were adopted for each channel, and we employed <5 kΩ impedance levels for reference electrodes on both mastoids.

### Data Analysis

Data were analyzed using Brain Electrical Source Analyses 6.0 (BESA; MEGIS Software GmbH, Gräfelfing, Germany). EEG data was re-referenced against linked mastoids. Filter and data cleaning parameters are based on analyses of the German experiment (Eulitz and Lahiri, 2004). Data was band pass filtered with low cutoff at 0.1 Hz (6 dB/oct slope) and high cut off 30 Hz (12 dB/oct slope). Epochs containing large non-eye artifacts found by visual inspection were discarded. Independent component analysis (ICA) was performed to correct for eye movement and blink artifacts. Remaining epochs with artifacts exceeding 100 μV within the −100 till 650 ms time window were discarded.

Each vowel category served as a standard in two blocks, for example: /e/ occurs as a standard in a block with [o] as a deviant as well as in a block with [ø] as a deviant. The first standard stimulus in a block was removed, as well as the first standard immediately after a deviant stimulus. Visual inspection showed that the event-related potential (ERP) waveforms of the same standard vowel in different blocks were completely overlapping in the 100–250 ms MMN time-window. Therefore, for standard vowels, the ERP was calculated over both blocks for every vowel. This way, we have the most robust measure for standards.

Each vowel has an inherent vowel specific auditory response due to its frequency characteristics, which is always there regardless of context. Event-related brain responses to different vowels show different waveform morphologies that may have nothing to do with any change detection process. The MMN response is a difference waveform between two ERPs (standard – deviant). To avoid effects due to the use of different vowels, the MMN is calculated by subtracting the ERP to a vowel when it is a deviant in a particular context (i.e., the vowel that is the standard in that block), from when that same vowel serves as a standard itself – so in a different block. For example, the MMN for the condition where [e] serves as a deviant and /o/ as standard is calculated as follows:

MMN[e]/0/=[ERPtostandard/e/]-[ERPtodeviant[e]incontextof/o/]

As such, the MMN reflects *change* perception only, and we can assess the impact of the context of the deviant. We calculated MMNs for each condition for each individual participant. For each experimental condition, we used two dependent variables for our analyses, which are similar to the ones used in Eulitz and Lahiri (2004):

1. *MMN latency* measured at the negative maximum amplitude at frontal electrode (Fz) in the latency range from 100 to 250 ms post-stimulus onset, based on a window around the mean MMN latency over all conditions.
2. *MMN amplitude (μV)* at Fz position measured as the mean amplitude across 80 ms centered at the mean MMN latency across subjects in the corresponding experimental condition.

These two parameters were subjected to a two-way repeated measures ANOVA. The ANOVA was restricted to the two pairs of inversion with a similar acoustic change: /e, ø/ and /o, ø/. The third vowel pair /e, o/ shows markedly larger acoustic difference between the two vowels (see Figure 2), and differed on two phonological features instead of one, and was therefore statistically tested separately. The ANOVA had two within-subject factors:

1. *Pair-of-Inversion* showing a similar acoustic change: [e]/ø/ versus [ø]/e/ and [o]/ø/ versus [ø]/o/.
2. *Direction-of-Change* of F2 frequency between standard and deviant: ascending in [e]/ø/ and [ø]/o/, but descending in [ø]/e/ and [o]/ø/.

This ANOVA assesses whether the MMN differs between vowel pairs (Pair of Inversion) and whether there are general acoustic asymmetric influences on the MMN (Direction-of-Change), or different asymmetries for different vowel pairs (interaction Pair-of-Inversion and Direction-of-Change).

Asymmetries in the MMN for the different vowel pairs were subsequently assessed by directly comparing the MMN characteristics (latency and amplitude at Fz) for the different directions of change by means of paired samples *t*-tests (planned comparisons).

## Results

**FIGURE 3:**
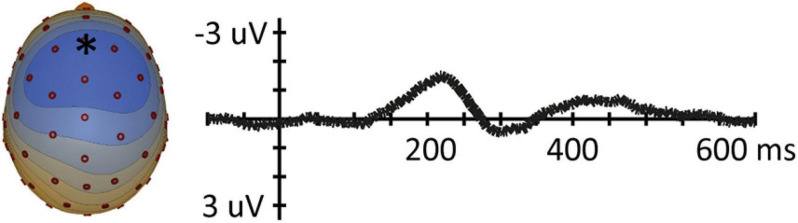
**(Left)** Overall MMN voltage topography map (μV) for the 5 ms time-window around the average MMN peak latency (218 ms). Blue in topoplot indicates negative potential, red positive potential. **(Right)** MMN waveforms at frontal electrode position (Fz).

A clear grand average overall MMN was found with a peak latency of 218 ms, with a clear frontal topography. Amplitude maps as well as voltage topography maps show typical MMN topographies (e.g., Näätänen, 2001; Näätänen et al., 2007) as is displayed in Figure 3 – with a predominant influence of left and right hemispheric temporal generators on the MMN, similar to Eulitz and Lahiri (2004). Visual inspection of grand average MMN waveforms showed a later peak latency than reported in Eulitz and Lahiri (2004), where all conditions had an MMN peak latency shorter than 170 ms, possibly due to the fact that we used a longer ISI. Because our grand average MMN was later, we used the time window of 100–250 ms to find peak latency values for each condition in each participant, covering the entire window of where a typical MMN peak would occur.

The two-way ANOVA revealed statistically significant interactions for *Pair of Inversion^∗^Direction of Change* for MMN amplitude at Fz (*F*(1,16) = 26.3; *p* < 0.001) as well as for MMN peak latency at Fz (*F*(1,16) = 6.3; *p* = 0.023). In addition, a main effect for *Pair of Inversion* (i.e., vowel pair) appeared significant (*F*(1,16) = 12.09; *p* = 0.003) for amplitude, but not for latency. *Direction of Change* appeared non-significant in both amplitude and latency measures.

The attested main-effect of *Pair of Inversion* in Fz amplitude shows that the two vowel pairs with similar acoustic distance behave differently. The lack of a main effect of *Direction of Change* implies that results cannot be explained merely on acoustic change in F2. The attested interaction in all measures implies that the impact of direction of change differs for different contrasts in processing of these vowel pairs.

**FIGURE 4:**
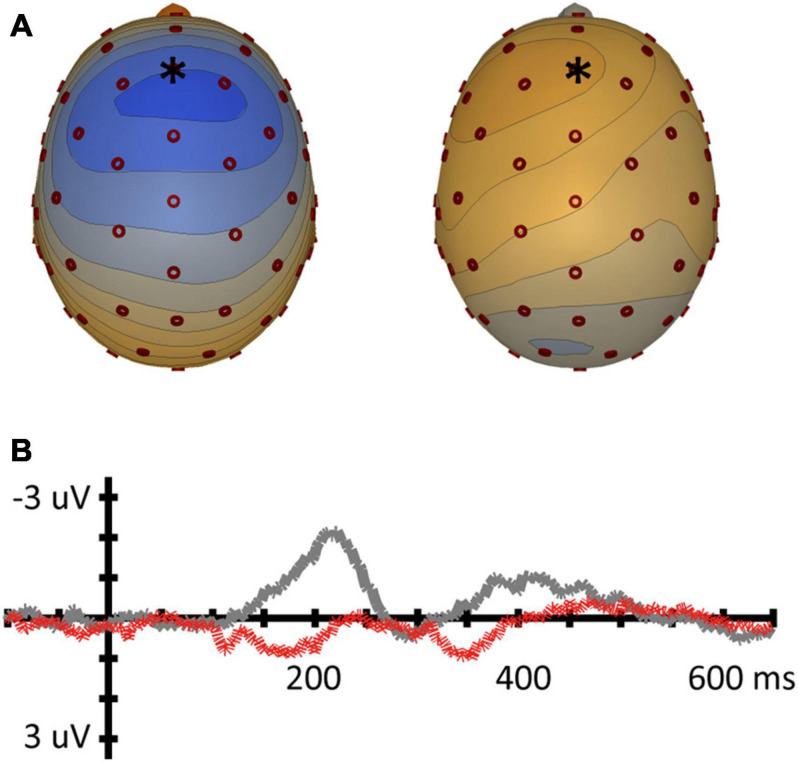
Mismatch negativity for the place contrast (vowel pair /o-ø/). **(A)** Topographic voltage plots of the MMN at the average MMN peak latency for both conditions (left: [ø]/o/, 210 ms; right [o]/ø/, 239 ms). Blue in topoplot indicates negative potential (μV), red positive potential, electrode Fz is indicated with an asterisk. **(B)** MMN waveforms for the Fz electrode [ø]/o/ (gray) and [o]/ø/ (red dashed).

**FIGURE 5:**
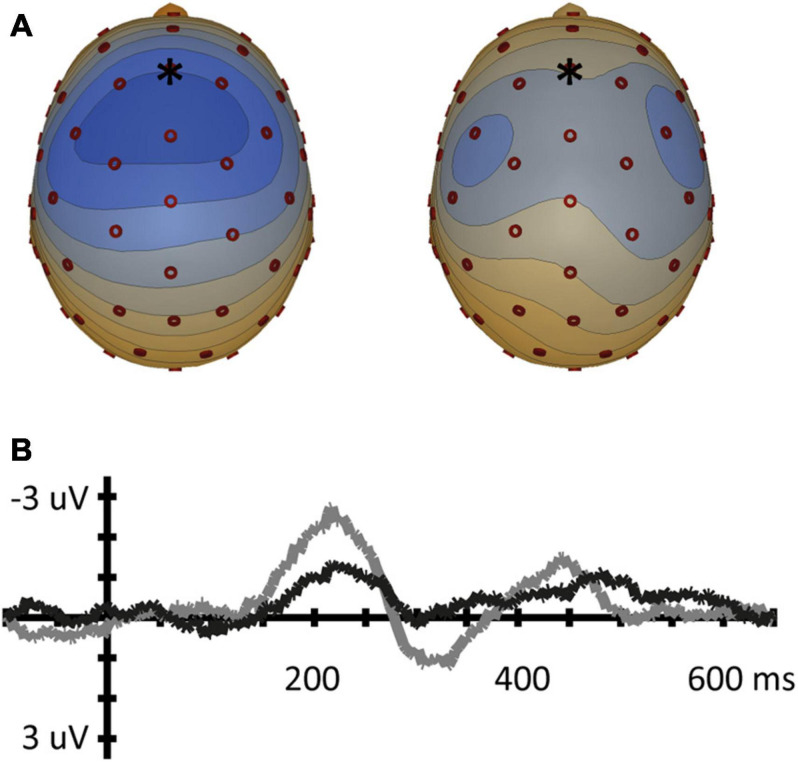
Mismatch negativity for the Labiality contrast (vowel pair /e-ø/). **(A)** Topographic voltage plots of the MMN at the average MMN peak latency for both conditions (left: [ø]/e/, 219 ms; right [e]/ø/, 216 ms). Blue in topoplot indicates negative potential (μV), red indicates positive potential, electrode Fz is indicated with an asterisk. **(B)** MMN waveforms for the Fz electrode [ø]/e/ (gray) and [e]/ø/ (black).

**FIGURE 6:**
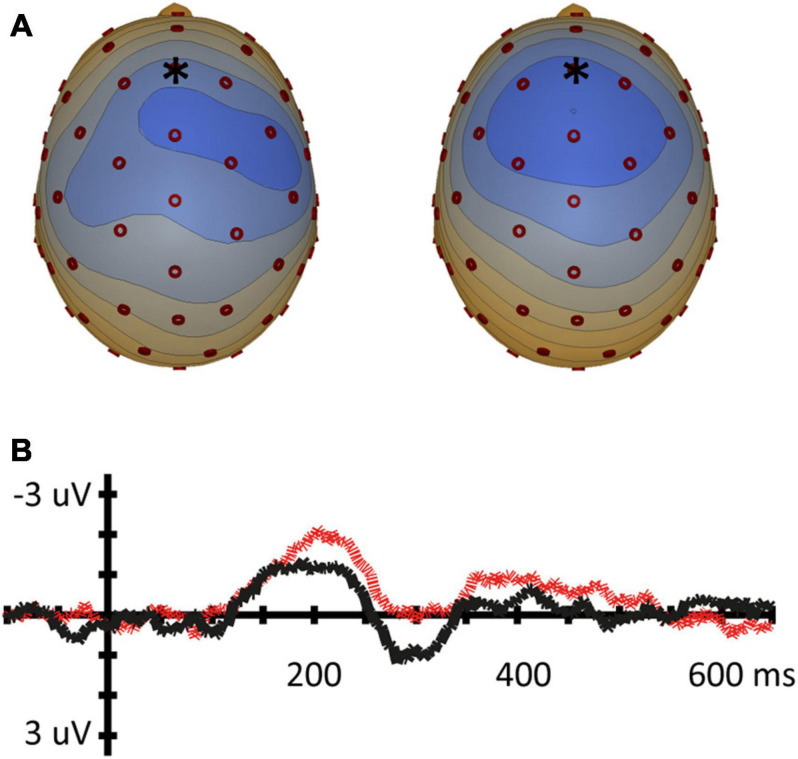
Mismatch negativity for the two-feature contrast (vowel pair /e-o/). **(A)** Topographic voltage plots of the MMN at the average MMN peak latency for both conditions (left: [e]/o/, 203 ms; right [o]/e/, 212 ms). Blue in topoplot indicates negative potential (μV), red indicates positive potential, electrode Fz is indicated with an asterisk. **(B)** MMN waveforms for the Fz electrode [e]/o/ (black solid line) and [o]/e/ (red dashed).

Planned comparisons of directions of change to assess asymmetries were tested with *t*-tests. Results of MMN amplitude and latency measured at Fz for each condition are reported in Table 2 below. MMN topographical plots and waveforms are presented in Figures 4–6 for each contrast separately.

**TABLE 2:** Table of results: MMN amplitude and peak latency at Fz for all six experimental condition.

	Vowel pair	Distinctive feature	Condition	Standard	Deviant	Mean Fz Amplitude ±SD (μV)	Mean peak latency ± SD (ms)
1	/o/-/ø/	Place	[ø]/o/	/o/	[ø]	−1.191.81*	21027*
			[o]/ø/	/ø/	[o]	0.281.31*	23938*
2	/e/-/ø/	Labiality	[ø]/e/	/e/	[ø]	−2.181.66*	21931
			[e]/ø/	/ø/	[e]	−1.081.28*	21629
3	/e/-/o/	Place & Labiality	[e]/o/	/o/	[e]	−1.490.93	20333
			[o]/e/	/e/	[o]	−2.061.1	21223

*Significance of paired-samples *t*-tests comparing directions within one vowel pair are indicated with an asterisk. Overview of experimental conditions: Vowels in square brackets [] represent the deviant vowel to which the MMN response is measured in a certain context. A vowel in // represents the standard (=context).*

For the place contrast /o, ø/, a paired *t*-test showed a significantly larger MMN amplitude at Fz for condition [ø]/o/ (*M* = −1.19 μV; SD = 1.81) than for condition [o]/ø/ (*M* = 0.28 μV; SD = 1.31); *t*(16) = 4.281, *p* < 0.001. The peak latency measure resulted in a significant asymmetry in the same direction: a shorter latency was found for [ø]/o/ (*M* = 210 ms; SD = 27) than for [o]/ø/ (*M* = 239 ms; SD = 38); *t*(16) = 2.823, *p* = 0.012.

For the labiality contrast /e, ø/, a paired *t*-test on Fz amplitude revealed a significantly larger MMN response for [ø]/e/ (*M* = −2,18 μV; SD = 1.66) compared to condition [e]/ø/ (*M* = −1.08 μV; SD = 1.28); *t*(16) = −4,053, *p* < 0.001. Thus, the results show a significant asymmetry between [ø]/e/ and [e]/ø/ with respect to amplitude. Fz MMN peak latency showed no significant difference between [ø]/e/ (*M* = 219 ms; SD = 31) compared to [e]/ø/ (*M* = 216 ms; SD = 29); *t*(16) = 0.284, *p* = 0.78.

For two-feature contrast /e, o/, the paired *t*-test did not show significant results: the MMN for [e]/o/ did not differ from the MMN for [o]/e/, neither in Fz amplitude ([e]/o/: *M* = −1.49 μV; SD = 0.93; [o]/e/ : *M* = −2.06 μV; SD = 1.1; *t*(16) = −1.857, *p* = 0.083), nor in Fz latency ([e]/o/; *M* = 203 ms; SD = 33; [o]/e/ (*M* = 212 ms; SD = 23; *t*(16) = 1.051, *p* = 0.309).

## Discussion

The present paper set out to test whether Dutch listeners show the same perceptual asymmetries as German listeners in an MMN paradigm in which three vowel pairs were investigated. We found significant asymmetries for both one-feature contrasts (place contrast /o, ø/, labiality contrast /e, ø/), but no significant asymmetry was found for our two-feature contrast (/e, o/). In section 5.1 we argue that the phonological specifications of the vowels together with the matching procedure can account for these differences. We discuss each pair separately. In section 5.2, we argue that acoustic differences between the vowels in each pair cannot straightforwardly explain the attested asymmetries. Before turning to the general conclusion, we also discuss predictions based on theories that rely on experience or frequency, and argue that these do not offer a clear explanation for the attested asymmetries.

### Explaining Asymmetries

#### Place of Articulation: /o/-/ø/

**FIGURE 7:**
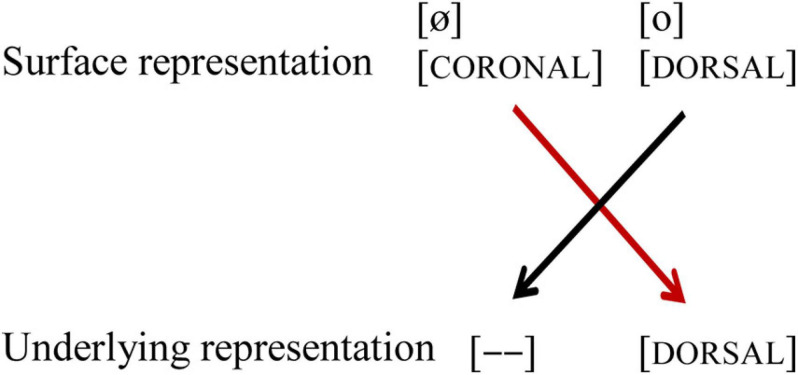
Asymmetrical perception of the vowel pair /o-ø/. Black lines indicate no-mismatch, the red line indicates a mismatch. [– –] indicates underspecification.

In the vowel pair /o/-/ø/, where the vowels share labiality, but differ in place of articulation, we replicated Eulitz and Lahiri (2004), in that a change from standard /o/ to deviant [ø] shows a larger MMN amplitude and shorter peak latency compared to the reverse [o]/ø/. In other words, there is a conflict in one direction. This provides evidence for [CORONAL] underspecification in Dutch, similar to what has been argued for German. A perceived [DORSAL] feature is a no-mismatch with an underspecified [CORONAL] feature, while a perceived [CORONAL] feature in the surface representation is a mismatch with a specified [DORSAL] feature in the underlying representation (see Figure 7). A similar finding has been found by De Jonge and Boersma (2015) for French, which also has a three-way contrast between the high vowels /i, y, u/ and mid vowels /e, ø, o/.

#### Labiality: /e/-/ø/

In the vowel pair /e/-/ø/, both vowels share place of articulation as both are [CORONAL]. They differ in labiality as /ø/ is specified for [LABIAL], while /e/ is not. The results from our study show a perceptual asymmetry: when /e/ is standard and [ø] deviant the data show a larger MMN amplitude than in the reverse condition [e]/ø/. This asymmetry was not reported for German. Eulitz and Lahiri (2004) argue that this is a non-conflicting situation with respect to place of articulation, which is why the MMN does not vary. In this condition, Dutch and German seem to differ. This finding raises at least two questions. First, how can we account for conflict based on surface and underlying features in the case of labiality, and second, why does this difference between the two languages arise, assuming the same underlying and surface features, as shown in Figure 1.

**FIGURE 8:**
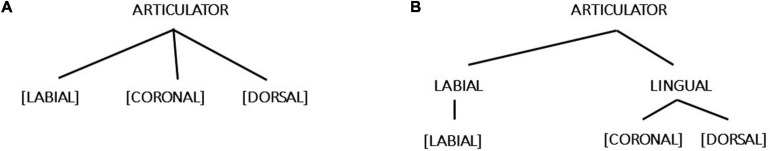
Relevant features [LABIAL], [CORONAL], and [DORSAL] in the feature tree of the FUL-model **(A)**, and the revised model proposed in this paper **(B)**.

In the FUL model, a mapping procedure is proposed, where features extracted from the acoustics are compared to features stored in the underlying representation. The goal of this feature mapping is to deselect unwanted candidates, and to limit the number of word candidates. Of course, only the relevant comparisons are made. After all, when for instance a feature [CORONAL] is extracted, only mapping to a mutually exclusive underlying feature like [DORSAL] would result in a meaningful/informative mismatch, whereas mapping [CORONAL] onto a feature like [HIGH] is neither efficient nor informative. In order for the mapping process to result in meaningful (no-)(mis)matches, the scope of the comparison of surface features must be defined. In the FUL model, [LABIAL], [CORONAL] and [DORSAL] share a node in the feature tree, the ARTICULATOR node, as shown in Figure 8A, an assumption shared with many others models (e.g., Chomsky and Halle, 1968; Sagey, 1986; Clements and Hume, 1995; Clements, 1999). Alternatively, [CORONAL] and [DORSAL] could be placed under a separate node, the LINGUAL node, as shown in Figure 8B. This assumption is also shared by various researchers (Browman and Goldstein, 1989; Keyser and Stevens, 1994). Keyser and Stevens (1994) assume [CORONAL] and [DORSAL] to form a single constituent – LINGUAL – because they both involve the tongue as its articulator. The tongue blade and tongue body are not completely independent, due to their anatomical connection. In contrast, the lips are anatomically and articulatorily independent from the tongue. For these reasons, Keyser and Stevens assume a separate LABIAL node, whereas [CORONAL] and [DORSAL] are parented by a LINGUAL node. Others have argued that features under the LINGUAL node often pattern together in phonological processes.

**FIGURE 9:**
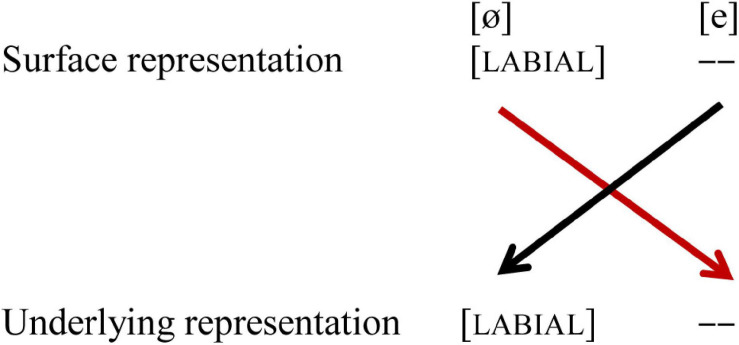
Asymmetrical perception of the vowel pair /e-ø/. Black lines indicate no-mismatch, the red line indicates a mismatch. [– –] indicates no feature selected (SR) and node lacking (UR).

For our purposes, separating a LABIAL and a LINGUAL node solves the issue that [LABIAL] may be combined with [DORSAL] and [CORONAL] (in languages like Dutch), which violates the mutual exclusivity assumption of FUL. With separate nodes for [LABIAL] on the one hand and [DORSAL] and [CORONAL] on the other hand, the mutual exclusivity assumption is no longer violated, since [LABIAL] no longer shares the parental node with [DORSAL] and [CORONAL]. This new hierarchy does not affect the place features discussed above, as [DORSAL] and [CORONAL] still share a node, but it does affect labiality as [LABIAL] is the only feature under the LABIAL node. A vowel is either [LABIAL] or lacks a LABIAL node. [NON-LABIAL] is neither a phonological feature, nor does it have stable acoustical features that would enable the perceptual system to extract it from the acoustics as a surface representation feature. When the acoustic feature corresponding to [LABIAL] is extracted from the signal and hence part of the surface representation, mapping it to the underlying representation with [LABIAL] results in a match. When the underlying representation lacks a LABIAL node, mapping cannot take place. This implies a phonological discrepancy between the surface and underlying representation, and we argue that such a case also indicates a phonological conflict. This is shown in Figure 9.

A similar asymmetry regarding the feature [VOICE] has been reported by Van der Feest and Fikkert (2015) in Dutch toddlers’ perception. They report that a change from a voiceless toward a voiced speech sound is perceptually more salient than vice versa, which resembles the case of labiality. A change from a non-feature (voiceless) toward a specified feature [VOICE] appears to result in phonological conflict, whereas the reverse does not. Lahiri (2018) also points out that [VOICE] could have its own node, similar to what we propose for [LABIAL]. This line of reasoning is also compatible with the perceptual asymmetries presented in Hestvik and Durvasula (2016) for English, albeit the specified feature here is [SPREAD GLOTTIS] for /t/, while /d/ is unspecified (lacking laryngeal specification). They noticed a larger MMN for [d]/t/ than for [t]/d/, although they presented a somewhat different analysis. Using a similar mapping rule, this would explain the laryngeal asymmetry (where either [VOICE] is specified as in Dutch, or [SPREAD GLOTTIS] as in English): the mapping cannot be completed when a voiced sound is heard but mapped onto a voiceless underlying representation where the VOICING node is absent, hence resulting in a phonological conflict. Whether this idea holds true more generally should be assessed in future research.

The second question raised by the results is why Dutch and German listeners’ brains react differently in this condition. Eulitz and Lahiri (2004) reported symmetrical perception for the vowel pair /e/-/ø/, which share place of articulation, and hence do not constitute a conflicting situation for place of articulation. This result was replicated for German listeners in an MMN passive oddball paradigm using both words and non-words (Cornell et al., 2011), who similarly report symmetrical results in this condition. This contrasts with the results in the current paper, which not only show a place asymmetry but also an asymmetry for labiality. One hypothesis is that the status of /ø/ as an underlying phoneme is different in Dutch and German. German has far fewer monomorphemic words with /ø/ than Dutch. In German, [ø] often arises as the result of morphological Umlaut as in the plural in *Vogel* [o] – *Vögel* [ø] “bird(s),” and in the comparative form of the adjective in *hoh* [o] – *höher* [ø] (“high – higher”). Hence, [ø] often occurs in derived environments, rather than in the lexicon. Although /ø/ occasionally also occurs in non-derived (lexical) environment, its contrastive value in German is limited, in comparison to Dutch, which may explain why German and Dutch listeners react differently to [LABIAL] in the context of [CORONAL] vowels: in German [LABIAL] is not a strong lexical contrast, while in Dutch it is. The implication is that the vowel inventory is based on lexical stems, rather than on all surface forms, and that hearing a front rounded vowel in German immediately activates the morphology. A perceived [LABIAL] will in those cases automatically activate back vowels in German, which is not the case in Dutch, where [LABIAL] is used to mark the contrast between /e/ and /ø/. While testing this is beyond the scope of this paper, it certainly warrants further research. There is some evidence in the literature that listeners are sensitive to critical phonological properties in parsing morpho-phonological forms (e.g., Post et al., 2008; Pliatsikas et al., 2014).

Scharinger (2009) and Scharinger et al. (2010) present an alternative approach to morphologically related words and assume that the vowels in lexical words that alternate in derived forms (i.e., certain morphological categories), such as the German word *Vogel*, have a different underlying representation than words with dorsal vowels that do not alternate. Specifically, they argue that the /o/ in *Vogel* is underspecified for [DORSAL], but specified for [LABIAL]. Under this view [ø] does not mismatch with the /o/ vowel in *Vogel*: the perceived [CORONAL] no longer mismatches with [DORSAL], as [DORSAL] is not part of the underlying representations. /ø/ and /o/_alternating_ have the same underlying representation, i.e., [LABIAL], but at a later stage a default coronal fill-in rule and a specific dorsal fill-in rule apply, differentiating both vowels in the surface representation. In terms of the hierarchy proposed in this paper, this would mean that these vowels lack specification under the LINGUAL node in the underlying representation. The influence of morphological alternations on the underlying vowel system requires further investigation.

#### Two Feature Contrast: /e/-/o/

The vowel pair /e/-/o/ differs in two features, both place of articulation and labiality. This vowel pair is also acoustically more different than both /e/-/ø/ and /o/-/ø/. We found no significant asymmetry for this two-feature contrast. From the discussion of the two vowel pairs with one-feature contrast, this may not come as a surprise. For the place contrast, we would expect an enhanced MMN in the context [e]/o/, but not vice versa. However, for the labiality contrast, we would expect an enhanced MMN in the context [o]/e/, as shown in Figure 9.

**FIGURE 10:**
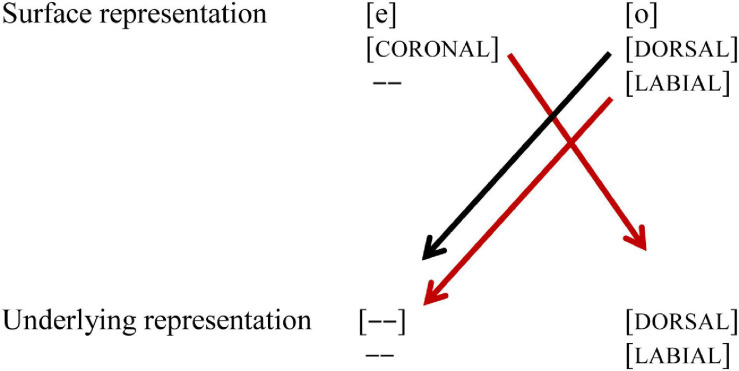
Asymmetrical perception of the vowel pair /e-o/. Black lines indicate no-mismatch, the red line indicates a mismatch. [– –] indicates no feature selected (SR) and node lacking (UR). The matching for place of articulation and labiality shows conflicting results.

One explanation for the lack of a significant asymmetry may thus be that these cancel each other out (see Figure 10). Another explanation could be that the two vowels are acoustically very different and easy to discriminate, as assumed in Eulitz and Lahiri (2004).

### Alternative Explanations

The literature mentions at least two other types of explanations for attested asymmetries in vowel perception: phonetic saliency and frequency of use. Phonetic saliency or ease of discrimination has been used to account for asymmetries in vowel perception, among others by proponents of the Natural Referent Vowel (NRV) framework (Polka and Bohn, 2011; Masapollo et al., 2017a,b). It assumes that vowels with formant frequencies closer together have focalized energy, and hence, universally are more salient in perception than vowels with formants further apart. The most focalized vowels are /i/, /a/ and /u/, the cornerstones of the vowel space. Consequently, changes from less to more focal vowels are easier to discriminate. In light of our study, a larger MMN is expected when the standard is non-focal, and the deviant is focal. Based on the convergence or closeness of F2 and F3, /e/ is more focal than /ø/, although toward the end of the vowel the difference becomes small, and the formants are slightly overlapping, making them less distinguishable. Based on the F1-F2 dimension, /ø/ would be more focal than /e/, but the formants do not get close, and it remains the question whether the formants lead to focalized energy. Moreover, this latter prediction is contradicting the claim made in Polka and Bohn (2011). In other words, the predictions based on the NRV are not entirely straightforward. For the vowel pair /o/-/ø/ the predictions are not clear either. Based on F1-F2, /o/ is more focal than /ø/. Based on F2-F3 /ø/ is more focal than /o/. The predicted asymmetries can be seen in Table 3.

**TABLE 3:** Overview of predictions for FUL, NRV and relative frequency.

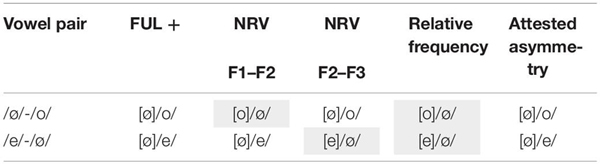

*For each *vowel pair* two conditions are tested in order to test two directions of discrimination. For each vowel pair and for each theory, the table indicates the direction of change in which the largest MMN is expected. During testing, surface features of the deviant [] are mapped onto the underlying features of the standard //. The predictions are conform the asymmetries in this paper, but the NRV makes predictions depending on closeness or convergence (based on F1-F2, as is the case /o/, or on F1-F2 or F2-F3, as in the case of /e/, as can be seen in Supplementary Appendix 3). The frequency predictions do make the opposite predictions. Marked in gray are the predictions that are not conform to our experimental findings, which are given in the final column.*

For frequency, or experienced-based theories, like for instance the Native Language Magnet (NLM) theory (Kuhl, 1991; Kuhl et al., 2008) it is usually assumed that category building is based on distributions in the input, and more frequent vowels have a stronger magnet effect, warping the perceptual space around them. Therefore, poorer discrimination is expected in the direction from more frequent to less frequent. However, this discrimination effect usually holds for within-category discrimination. If both are categories in the language, predictions are less clear. However, one could hypothesize that frequent vowels allow for more variation, and hence it is expected that a frequent standard and an infrequent deviant would be more difficult to discriminate, and hence show a smaller MMN effect, than vice versa. With respect to frequency, front round vowels are relatively infrequent in Dutch compared to front (unround) vowels and back (round) vowels. Baayen et al. (1995) report the following percentages for the relevant vowels in CELEX: [e] = 6.7%; [o] = 6.0%; [ø] = 2.3%, meaning /e/ and /o/ occur roughly twice as often as /ø/ in Dutch. The predictions based on these vowel distributions are thus opposite to the findings in our experiment. The different predictions are presented in the Table 3.

To summarize, the current NRV framework only partly predicts the attested asymmetries. It must be noted that the results reported in Polka and Bohn (2011) do not always conform to their own predictions. Notably, they report better discrimination in the direction from/ e/ to /ø/ in Danish infants. The frequency account does not make the right predictions, and it also raises questions as how large the frequency difference needs to be to predict asymmetries. The proposed geometry and mapping algorithm in this paper shows how listeners might evaluate the incoming signal based on its phonology, which is conform the attested asymmetries.

## Conclusion

The current study showed evidence for asymmetrical processing of vowels, and the attested asymmetries provide further support for the FUL model, in which underlying phonological representations are underspecified. It replicated the place asymmetry in German listeners as reported in Eulitz and Lahiri (2004) for Dutch. While they did not find asymmetries between /e/ and /ø/, i.e., asymmetries based on [LABIAL], this asymmetry was shown in the current study. We propose to place [LABIAL] under a separate node from [CORONAL] and [DORSAL] (Figure 8B), which we group together under a LINGUAL node, following for instance Keyser and Stevens (1994). Our proposal has at least two benefits: features under the same node are mutually exclusive, a central assumption made by FUL, which however had to be relaxed to allow [LABIAL] to combine with [CORONAL] or [DORSAL] in the traditional FUL model. Moreover, as [LABIAL] is the only feature under the LABIAL node, its mapping must match, as a no-mismatch is no viable option. If the perceived [LABIAL] in the surface representation cannot be matched, the mapping is aborted, leading to a conflict, and hence a perceptual asymmetry. This is the case when the standard is /e/ (no labial node), and the deviant [ø], with a surface feature [LABIAL]. A consequence is that not only a mapping mismatch (as traditionally discussed in FUL), but also an aborted mapping implies a phonological conflict. Finally, this study shows that languages can show cross-linguistic differences if contrasts are implemented differently in the languages.

## Data Availability Statement

The raw data supporting the conclusions of this article will be made available by the authors, without undue reservation.

## Ethics Statement

The studies involving human participants were reviewed and approved by “Commissie Mensgebonden Onderzoek” (CMO) Arnhem-Nijmegen, Netherlands, under the general ethics approval (Imaging Human Cognition, CMO 2014/288). The patients/participants provided their written informed consent to participate in this study.

## Author Contributions

NR and PF: conceptualization, writing – original draft preparation, writing – review and editing, and project administration. NR and TS: methodology, software, validation, formal analysis, and visualization. NR: investigation and data curation. PF: resources and funding acquisition. TS and PF: supervision. All authors have read and agreed to the published version of the manuscript.

## Conflict of Interest

The authors declare that the research was conducted in the absence of any commercial or financial relationships that could be construed as a potential conflict of interest.
