# Supplementary material for: Contrast and Conflict in Dutch Vowels
Source: Front Hum Neurosci. 2021 Jun 7;15:629648. doi: 10.3389/fnhum.2021.629648 (PMC8215709; doi:10.3389/fnhum.2021.629648)
Supplement: Supplementary file 1 [file Data_Sheet_1.docx]

Appendix 1: Stimuli creation

Here we provide a brief description of how the stimuli were created. Before using the stimuli in the EEG-experiment, three pretests (see Appendix 2) were performed to evaluate and adjust the stimuli.

**Recordings**

A male speaker (age 28) produced the three different vowels (/e/, /o/, and /ø/) in a set (i.e. triad) in both /V/ (where V stands for *vowel*) and /hV/ context (e.g. /e, o, ø/, or /he, ho, hø/). A difference in context does not make a difference for the vowels’ place of articulation features. The voiced consonant /h/ is articulated with glottal friction, with the same shape of vocal tract as that of the following vowel (e.g. Booij, 1995). Already during pronunciation of the /h/, the oral cavity adopts the shape to produce the subsequent V.

The speaker was instructed to pronounce all vowels with similar and steady pitch, and similar duration. We used triads to facilitate similar pitch, duration and loudness features for the different vowels. However, when our speaker pronounced a triad, he would produce the first syllable with rising pitch, and the third syllable with falling pitch. Even after extensive and explicit instruction this list intonation still appeared in his speech. We resolved this issue by having him produce triads in which the order of vowels differed, (e.g. /e o ø/ /o ø e/, and /ø e o/ and similarly for hV context). The syllable in the middle received a relatively constant pitch, and all three vowels appeared in this position.

**Stimuli preprocessing**

All preprocessing steps were conducted in PRAAT (version 5.3.22). Tokens of the recorded vowels were selected based on similarity in pitch (within and between category) and formant contours (within category), resulting in three most similar tokens of each vowel category. Eventually, all tokens that were selected for the experiment of /o/, and /ø/ originated from a hV syllable, while all /e/ tokens originated from a V context.

Selected vowels were cut to have a 200 ms duration. Because vowels’ features at their centre are most representative for their specific vowel category (Rietveld & van Heuven, 2001), the midpoint relative to the duration of the original vowel served as the midpoint for extraction of the 200 ms portion. For all vowels, 200 ms was extracted; for none of the stimuli original vowel initiation was preserved. Before determining the midpoint of the vowel, /h/ was removed for the /o/ and /ø/ tokens. For hV syllables, /h/ was removed before further steps were carried out. Of the resulting tokens, mean amplitude was scaled at 70 dB, and amplitude ramps of 50 ms were applied on both onset and offset using the Audio Manipulation script by Kerkhoff (2009).

Appendix 2: Pretests of stimuli

Before using the stimuli in neuroimaging experiments, their quality was determined in three behavioral pretests.

- *Pretest 1* assessed whether differences in original vowel context (either /hV/ or /V/ context) was still audible. To use the stimuli in subsequent EEG-experiments, no audible difference between contexts was required.
- In *Pretest 2* we controlled for perceptual loudness differences for the three different vowel categories. Perceptual loudness differences might occur as a result of differences in characteristic formant frequencies for the different categories.
- In *Pretest 3* we evaluated perceptual variability between the tokens within each vowel category. To use the stimuli in subsequent EEG-experiments, no difference in within-category variation should be perceived.

**Participants**

Pretest 1 and Pretest 2 were performed by two adult male subjects (both age 28) and were performed in a quiet room. Both subjects participated on voluntary basis. Pretest 3 was performed by 16 adult participants (50% females) (ages ranging between 20 and 29; M = 24.6; SD = 3.3) in a sound attenuated booth. Participants of Pretest 3 were reimbursed for their participation: 5 Euro or 0,5 study credits. All participants in all pretests were right-handed adult native Dutch listeners, consistent with the participants eligible for participating in the eventual EEG-experiment. They had no background in linguistics, and reported normal hearing, and no speech or language impediments. No dialect speakers were included.

**PRETEST 1: Original context: /hV/ vs. /V/**

In Pretest 1 we assessed whether the original context of the stimuli was still audible. All tokens of /o/ and /ø/ originated from an hV context, while all /e/ tokens had a V context. This difference in original context should not be detectible in our stimuli. To verify this, we randomly presented all 9 individual stimuli on a laptop (HP Probook 6750B) using Presentation Software (version 18.2 02.18.16, [www.neurobs.com](http://www.neurobs.com)). All stimuli were presented 3 times, resulting in 27 trials. Subjects judged whether they heard /V/ or /hV/, responding by respectively pressing keys [1] or [2] on the keyboard. Stimuli were auditorily presented through over ear headphones (Sennheiser HD 215) at fixed volume.

Participants were unable to detect context above chance level. Participants reported that all trials just seemed to be /V/, and they did not hear the initial /h/. In 33% of /o/ and /ø/ trials participants selected /hV/ context, which is considerably below chance level of 50%. Because no obvious /hV/ stimuli were presented, participants became motivated to press /hV/ also in trials where they heard no obvious /h/. This has probably fed /hV/ responses. Participants were not consistent in their judgment, selecting /hV/ at one trial, and /V/ at another trial for identical stimuli. We concluded that, as we expected, the initial /h/ was successfully deleted from the signal as /h/ was no longer perceivable.

**PRETEST 2: Perceptual loudness**

Due to non-linear frequency response characteristics of the human auditory system, different frequencies with matched amplitude (objective loudness in dB) do not necessarily have similar perceptual loudness. For example, low frequency sounds are perceptually louder than high frequency sounds. These effects differ at different intensities. The stimuli are to be used in Mismatch Negativity EEG-experiments. To avoid mismatch negativity responses driven by differences in perceptual loudness between the three vowel categories of /e/, /o/ and /ø/ in the EEG-experiments, we controlled for perceptual loudness following the procedure described below. In Pretest 2 we assessed whether the stimuli were similar in perceptual loudness. Different vowel categories have different frequency characteristics.

In PRAAT (version 5.3.35; Boersma & Weenink, 2012) we normalized all stimuli to have a matching RMS amplitude of 70 dB. We ran a judgment task on a laptop (HP Probook 6750B) using Presentation Software. In this AX-task, all possible stimulus pairs were presented to the participants. Stimuli were presented through headphones (Sennheiser HD 215). Each stimulus was compared to each of the other stimuli (resulting in within-category trials and between-category trials), but never to itself, thus resulting in 9*8 = 72 trials, presented in random order. Subjects judged whether stimulus X (= the second stimulus of a trial) was less loud, equally loud, or louder than stimulus A by choosing respectively keys [1], [2] or [3].

Subjects reported that /e/ stimuli sounded louder than the other two vowel categories. The button-press data support this judgement. Surprisingly, this perceptual difference appeared asymmetric. In 71,9% of trials with /e/ presented last (stimulus X) and another vowel category as stimulus first (stimulus A), /e/ was considered louder. However, in the reversed situation where A = /e/ and X was any token of the other two vowel categories, /e/ was considered louder in only 9,4% of the trials. This implies a recency effect. A difference in perceptual loudness can therefore be considered small. In only 2,7% (= 4 cases), a non-/e/ token was considered louder than another token. Only once (<1%) an /e/ sound was considered softer than a stimulus of the other two vowel categories.

Relative loudness of sounds in air as perceived by the human ear are expressed in dBA. Perceptual loudness of all stimuli was measured using a dB(A) meter, measuring loudness as produced by headphones. Consistent with the results of the judgment task, the /e/ sounds resulted in slightly higher dBA values (+/- 67 dBA for /e/ and +/- 64 dBA for the other two categories) despite identical dB. Hence, amplitude of /e/-stimuli was attenuated in PRAAT until dBA-values of /e/ tokens were similar to the other two vowel categories. As a result, all tokens of all categories were within a 2 dBA range with a mean of 64 dBA. In all following experiments, volume was set to achieve this same dBA intensity, thus controlling for perceptual loudness.

**PRETEST 3: Within-category variation**

After the intensity adjustments as mentioned in Pretest 2 we proceeded with a more thorough pretest. In this third pretest we assessed whether all three vowel categories comprise similar degrees of within-category variation.

By using several natural tokens of each vowel, acoustic within-category variability is introduced. This simulates more natural speech perception conditions and – most importantly – forces a listener’s processing system to map the incoming acoustic signals onto more abstract representations (Eulitz & Lahiri, 2004). In other words, subjects will listen in a categorical way rather than focus on minor acoustic differences between tokens. Thus, some within-category variation is desired. However, for each category (/o/, /e/ and /ø/) we aim for similar degrees of within-category variability. If there is a particular token within a category that stands out, this could lead to within-category elicitation of a Mismatch Negativity response and could thus have an undesirable influence on the overall category MMN response. Differences in MMN for the different vowel categories should not be due to differences in within-category variation. Thus, in Pretest 3, we tested whether the perceptual variation within the vowel categories was similar for all three vowel categories.

We performed an ABX task (or *matching-to-sample* task); a discrimination procedure where triads of stimuli are presented. The first two (A and B) are references and the third/last stimulus (X) is compared to these references. Subjects were instructed to determine whether the third vowel (stimulus X) in a triad was more similar to either the first (stimulus A) or the second (stimulus B) sound. They responded by pressing button [1] for stimulus A or [2] for stimulus B on the computer’s keyboard. Stimuli were presented through headphones in a sound attenuated booth. Volume was set at 64 dBA for all stimuli. Participants were not allowed to adjust the volume. Inter-stimulus-interval (ISI) was set at 700 ms, similar to the eventual EEG-experiments. Before the start of the experiment participants were informed about the experiment and signed a consent form.

The ABX-experiment was run using Presentation Software. Within each block, trials were presented in random order. The experiment started with 6 practice trials with between-category trials. Then, 3 test blocks of 54 within-category trials (e.g. A = [e1] - B = [e3] - X = [e2]) were presented (PART 1). Each individual triad occurred in each of the three blocks. In addition, a final test block (PART 2) was included (36 trials), containing only between-category trials (e.g. A = [e1] - B = [o2] - X = [o3]). The three blocks of within-category trials (PART 1) can show the degree of within-category variation. PART 2, testing between-category trials, can provide proof that participants can behaviorally discriminate the vowels of different categories. In between blocks subjects were free to take breaks. After the experiment, subjects filled out a post-test questionnaire regarding strategy use, difficulty, and assumptions with respect to research questions.

Only responses with *100 > RT < (average RT + 2SD)* were included in the data assessment.

Results showed that participants were able to discriminate between vowel categories /e/, /o/, and /ø/ (92,5% correct) (PART 2 in the experiment).

In PART 1 we can distinguish two conditions: (1) *Correctness*_*Condition*: condition where X is identical to A or B and a correct/incorrect answer can be defined, and (2) *Preference*_*Condition*: condition where X is not identical to either A or B. Thus, no correctness can be assessed. The latter condition provides information about the degree of similarity of X to A or B and thus provides info about the within-category variation. As we expected, identifying a stimulus in a within-category trial (Correctness Condition in PART 1) was close to chance (56,6% correct). In the Preference Condition, option A was selected 51% of the time as opposed to 49% for option B. This is a very close approximation of random ratio. Within each vowel category, random responses would lead to 1/3 of responses for each token. Results were close to this ratio (see Table A2.1). These ratio’s did not deviate enough from 33,3% to conclude that there were salient differences between the tokens within each category.

**Table A2.1**. Response ratio’s to each token of each vowel category in PART 1 of the ABX-pretest.

|  | /e/ | /o/ | /ø/ |
| --- | --- | --- | --- |
| token 1 | 36% | 30% | 36% |
| token 2 | 23% | 36% | 30% |
| token 3 | 41% | 34% | 34% |

Appendix 3: Stimuli characteristics

**Table A3.1**. Fundamental frequencies (Hz) of all tokens used as stimuli

| Vowel | F0 Token1 | F0 Token2 | F0 Token3 | Mean F0 |
| --- | --- | --- | --- | --- |
| /e/ | 111.8 | 112.7 | 113.0 | 112.5 |
| /ø/ | 113.4 | 114.3 | 114.1 | 113.9 |
| /o/ | 112.0 | 113.2 | 112.8 | 112.7 |

**Table A3.2**. Formant frequencies (Hz) for formants F1, F2 and F3 of three tokens of /e/. Measures sampled at 25%, 50% and 75% of vowel. The averaged values for formant frequencies are taken at the 20-30% interval (t=25%), 45-55% (t=50%) interval and 70-80% (t=75%) interval. *t =* time point. Each vowel had a duration of 200 ms. As a reference, average formant frequencies as reported in Adank, van Hout, and Smits (2004) for Northern standard Dutch by male speakers are included.

|  | **Token [e]1** | | | **Token [e] 2** | | | **Token [e] 3** | | | **Adank et al. /e/** | | |
| --- | --- | --- | --- | --- | --- | --- | --- | --- | --- | --- | --- | --- |
| ***t*** | **25%** | **50%** | **75%** | **25%** | **50%** | **75%** | **25%** | **50%** | **75%** | **25%** | **50%** | **75%** |
| **F1** | 517 | 458 | 336 | 415 | 400 | 361 | 472 | 472 | 354 | 541 | 348 | 303 |
| **F2** | 1822 | 1881 | 1951 | 1887 | 1995 | 2075 | 1847 | 1877 | 1928 | 1901 | 1977 | 2013 |
| **F3** | 2230 | 2341 | 2482 | 2545 | 2583 | 2597 | 2254 | 2347 | 2498 | 2416 | 2518 | 2600 |

**Table A3.3**. Formant frequencies (Hz) for F1, F2 and F3 of three tokens of /ø/. Measures sampled at 25%, 50% and 75% of vowel. The averaged values for formant frequencies are taken at the 20-30% interval (t=25%), 45-55% (t=50%) interval and 70-80% (t=75%) interval. *t =* time point. Each vowel had a duration of 200 ms. As a reference, average formant frequencies as reported in Adank, van Hout, and Smits (2004) for Northern standard Dutch by male speakers are included.

|  | **Token [ø]1** | | | **Token [ø]2** | | | **Token [ø]3** | | | **Adank et al. /ø/** | | |
| --- | --- | --- | --- | --- | --- | --- | --- | --- | --- | --- | --- | --- |
| ***t*** | **25%** | **50%** | **75%** | **25%** | **50%** | **75%** | **25%** | **50%** | **75%** | **25%** | **50%** | **75%** |
| **F1** | 384 | 365 | 320 | 407 | 375 | 329 | 409 | 337 | 296 | 396 | 344 | 295 |
| **F2** | 1432 | 1535 | 1607 | 1544 | 1563 | 1592 | 1475 | 1573 | 1588 | 1472 | 1594 | 1591 |
| **F3** | 2123 | 2104 | 2151 | 2254 | 2241 | 2229 | 2126 | 2170 | 2070 | 2125 | 2174 | 2072 |

**Table A3.4**. Formant frequencies (Hz) for F1, F2 and F3 of three tokens of /o/. Measures sampled at 25%, 50% and 75% of vowel. *t =* time point. The averaged values for formant frequencies are taken at the 20-30% interval (t=25%), 45-55% (t=50%) interval and 70-80% (t=75%) interval. Each vowel had a duration of 200 ms. As a reference, average formant frequencies as reported in Adank, van Hout, and Smits (2004) for Northern standard Dutch by male speakers are included.

|  | **Token [o]1** | | | **Token [o]2** | | | **Token [o]3** | | | **Adank et al. /o/** | | |
| --- | --- | --- | --- | --- | --- | --- | --- | --- | --- | --- | --- | --- |
| ***t*** | **25%** | **50%** | **75%** | **25%** | **50%** | **75%** | **25%** | **50%** | **75%** | **25%** | **50%** | **75%** |
| **F1** | 530 | 420 | 294 | 444 | 412 | 365 | 527 | 420 | 289 | 524 | 401 | 252 |
| **F2** | 1013 | 976 | 877 | 1015 | 929 | 848 | 1021 | 990 | 881 | 1017 | 968 | 892 |
| **F3** | 2250 | 2230 | 2286 | 2258 | 2306 | 2371 | 2307 | 2237 | 2261 | 2249 | 2244 | 2272 |

Appendix 4: RMS data

Table A4.1. Results root-mean-square (RMS) MMN amplitude measure for six conditions.

| Vowel pair | Contrastive feature | Condition | RMS Amplitude ± SEM (μV) |
| --- | --- | --- | --- |
| /ø - o/ | place | [ø]/o/ | 1,15 ± 0,173 |
|  |  | [o]/ø/ | 0,77 ± 0,096 |
| /e - ø/ | labiality | [e]/ø/ | 0,93 ± 0,149 |
|  |  | [ø]/e/ | 1,48 ± 0,211 |
| /e - o/ | place & labiality | [e]/o/ | 0,95 ± 0,105 |
|  |  | [o]/e/ | 1,17 ± 0,137 |

An interaction of *Pair of Inversion * Direction of Change* was significant (F(1/16) = 7,46; p = 0.015. No main effect for *Pair of Inversion* was found: F(1/16) = 3.4; p = 0.08. Also no significant main effect was found for *Direction of Change:* F(1/16) = 0.53; p = 0.47. The found interaction implies that in processing of these vowel pairs, the impact of direction of change differs for different contrasts.

Apart from this ANOVA, we ran planned comparisons. For the place contrast /ø-o/, paired samples t-tests for the difference between [ø]/o/ (M = 1.15; SD = 0.71) and [o]/ø/ (M = 0.77; SD = 0.93) did not give significant results; t(16) = -1.96, p = 0.067.

For the labiality contrast /e-ø/, paired samples t-tests resulted in a significant asymmetry: t(16) = 2.59, p = 0.020, with a larger amplitude for [ø]/e/ (M = 1.48; SD = 0.62) than for [e]/ø/ (M = 0.93; SD = 0.87).

For the two-feature contrast /e-o/, paired samples t-tests for the for the difference between [e]/o/ (M=0.95; SD=0.43) and [o]/e/ (M=1.17; SD=0.57) did not give significant results; t(16)=-2.004, p = 0.062.
